# Relational development in children with cleft lip and palate: influence of the waiting period prior to the first surgical intervention and parental psychological perceptions of the abnormality

**DOI:** 10.1186/1471-2431-12-65

**Published:** 2012-06-08

**Authors:** Bruno Grollemund, Antoine Guedeney, Marie-Paule Vazquez, Arnaud Picard, Véronique Soupre, Philippe Pellerin, Etienne Simon, Michel Velten, Caroline Dissaux, Isabelle Kauffmann, Catherine Bruant-Rodier, Anne Danion-Grilliat

**Affiliations:** 1 Département d’Orthopédie Dento-Faciale, Pôle de médecine et chirurgie buccodentaires, Hospices civils, Place de l’Hôpital 1, Strasbourg, 67000, France; 2 Laboratoire éthique et pratiques médicales, IRIST EA 3424, Université de Strasbourg, Rue de l’Université 7, Strasbourg, 67000, France; 3 Service de Psychiatrie Infanto-Juvénile Hôpital Bichat Claude-Bernard, Groupement Hospitalier Universitaire Nord, Rue Henri Huchard 46, Paris, 75018, France; 4 CMP, Rue René Binet 64, Paris, 75018, France; 5 Service de Chirurgie Maxillo-Faciale, Hôpital d’Enfants Armand-Trousseau AP HP, Avenue du Docteur Arnold Netter 26, Paris Cedex 12, 75571, France; 6 Centre de référence des Malformations Cranio Maxillo Faciales Rares, Hôpital Salengro, CHRU de Lille, Lille, 59037 Cedex, France; 7 Service de Chirurgie Maxillo-Faciale et Plastique de la Face, Hôpital Central, Avenue du Maréchal de Lattre de Tassigny 29, Nancy Cedex, 54035, France; 8 Laboratoire d’épidémiologie et de santé publique, Faculté de médecine et Centre Paul Strauss, Rue Kirschleger 4, Strasbourg Cedex, 67085, France; 9 Service de Chirurgie Plastique et Maxillo-Faciale, Hôpitaux Universitaires de Strasbourg, Place de l’Hôpital 1, Strasbourg, 67000, France; 10 Service de Chirurgie Pédiatrique, Hôpitaux Universitaires de Strasbourg, Place de l’Hôpital 1, Strasbourg, 67000, France; 11 Service Psychothérapique pour Enfants et Adolescents, Hôpitaux Civils, Hôpitaux Universitaires de Strasbourg, Place de l’Hôpital 1, Strasbourg, 67000, France

**Keywords:** Cleft lip, Cleft lip and palate, Parental representation, Parenting stress, Prenatal diagnosis, Psychosocial

## Abstract

**Background:**

The birth of a child with a cleft lip, whether or not in association with a cleft palate, is a traumatic event for parents. This prospective, multidisciplinary and multi-centre study aims to explore the perceptions and feelings of parents in the year following the birth of their child, and to analyse parent–child relationships. Four inclusion centres have been selected, differing as to the date of the first surgical intervention, between birth and six months. The aim is to compare results, also distinguishing the subgroups of parents who were given the diagnosis *in utero* and those who were not.

**Methods/Design:**

The main hypothesis is that the longer the time-lapse before the first surgical

intervention, the more likely are the psychological perceptions of the parents to affect the harmonious development of their child. Parents and children are seen twice, when the child is 4 months (T0) and when the child is one year old (T1). At these two times, the psychological state of the child and his/her relational abilities are assessed by a specially trained professional, and self-administered questionnaires measuring factors liable to affect child–parent relationships are issued to the parents. The Alarme Détresse BéBé score for the child and the Parenting Stress Index score for the parents, measured when the child reaches one year, will be used as the main criteria to compare children with early surgery to children with late surgery, and those where the diagnosis was obtained prior to birth with those receiving it at birth.

**Discussion:**

The mental and psychological dimensions relating to the abnormality and its correction will be analysed for the parents (the importance of prenatal diagnosis, relational development with the child, self-image, quality of life) and also, for the first time, for the child (distress, withdrawal). In an ethical perspective, the different time lapses until surgery in the different protocols and their effects will be analysed, so as to serve as a reference for improving the quality of information during the waiting period, and the quality of support provided for parents and children by the healthcare team before the first surgical intervention.

**Trial Registration:**

ClinicalTrials.gov Identifier: NCT00993993.

## Background

### The malformation and its consequences

Cleft lip with or without cleft palate (CLP), or cleft palate alone (CP) are the most frequent cranio-facial malformations in humans. The prevalence in France is 1/700 births, but incidence varies according to geographical origins from 1/300 births for Asians to 1/2500 births among Africans [1-3]. Two clinical forms are generally distinguished: CP, and unilateral or bilateral CLP which account for 70 to 80% of cases.

The diagnosis of these malformations may be delivered either *in utero* from prenatal scans, or at birth. The consequences of CLP are both aesthetic and functional (phonation, hearing, swallowing, chewing and ventilation are affected); there are also psychological consequences (construction of a self-image, relational disturbances). Over the years, numerous studies have been performed on the psychological effects of CLP, both for the child and for the parents. One review of the literature [4] notes that these studies overall do not conclude to any major psychosocial problem. Individuals presenting a CLP do not appear to be clearly affected by psychological, and even less psychiatric, problems. However disturbances have been described: behavioural disturbances, anxiety, depression, aesthetic dissatisfaction with facial appearance, these being observed in both children and adults. The difficulty in interpreting these signs resides in the many factors liable to influence this type of condition (family environment, the size and type of the cleft, the surgical protocol, growth, the social environment etc. [5].

Careful attention to what families have to say in the course of the numerous appointments required for treatment brings to light the importance, for the development of the child affected, of the trauma experienced by parents when the malformation is discovered - often seriously affecting the facial appearance of the baby. It also focuses the importance of the psychological context within which the parent–child relationship becomes established. The early relationship of parents with their newborn child is based on conscious and unconscious emotions which for the most part are relayed by touch, tone of voice, looks, and facial expressions. When an infant has a CLP, the parents are unexpectedly confronted with a damaged, split-open face, and the emotional overload of an event of this sort can hinder their affective investment. Looking at their newborn child's deformed face obviously generates contradictory emotions: distress, horror, guilt, desire to mend or to protect and so forth [6]. The malformation can prevent recognition of an intergenerational affiliation, and hamper the integration of the child into the family [7]. Thus the parent–child relationship can be affected from birth.

### The surgical treatment of CLP

The care provision for children with CLP requires several surgical interventions depending on the seriousness of the malformation. The first intervention is on the lip, and a second, a few months later, is on the palate. The way in which the corrections are performed depends on the defect and the protocol chosen by the surgical team. To date, no generic protocol has been recognised by the medical community as a whole. Each patient is cared for according to the experience and specific choices of the team in charge. In Europe there are more than 210 referral centres for children with CLP, and some 190 different protocols. These disparities can be explained by the fact that the aesthetic and functional result of a protocol can only really be evaluated in adulthood, when the child's growth is complete. Yet the period between the diagnosis and the instatement of the protocol by way of the first surgical procedure is crucial, since it is in that period that the relationship between the newborn child and the parents becomes established. The malformation, particularly in the case of cleft lip which directly affects the child's face, is likely to affect the parents' attachment to the child.

In France, as elsewhere, the planning of the surgical intervention varies according to the care facility. Certain teams are in favour of early intervention, immediately after birth, so as to establish proper functioning (ventilation, swallowing and phonation) as soon as possible, and also to reduce the psychological impact of the defect on the parents and the family circle. Other teams prefer to wait three of even six months. By thus delaying the intervention, these teams aim to make use of the particularly fast growth during this period. The individualisation of the different muscles is thus easier, and this enhances the precision and the quality of the surgical gesture. For certain authors, these differences in timing could condition the child's cognitive development, ultimately influencing achievement in school [8,9]. These authors suggest that the disfigured faces of these children are less attractive, and also that it is difficult for parents to interpret their children's facial expressions. This is important in the first months of life. Murray et al in 2008 [10] showed interdependence between a child's cognitive development and the timing of the surgical intervention. Development appeared to be retarded in case of difficulties in relations between mother and child at the age of two months, and if the first intervention was planned at a later stage. In contrast, no difference with the control group was noted for the subgroup of children in this study for whom surgery was planned early. A disfigured child can affect the mother psychologically, and upset the relationship. In this case the length of time during which the child remains disfigured could explain the cognitive delay.

### The importance of informing, and instatement of care

All the studies on the early life of children with CLP underline the importance of the quality of information right at the start of the relationship with the care team, and at the time of the disclosure of the diagnosis. In 2004 Rey-Bellet and Hohlfeld [11] showed that a larger proportion of families deplored the lack of knowledge, know- how and tact among healthcare teams when the childbirth occurred in a peripheral maternity hospital rather than a large central hospital. When the diagnosis was prenatal, the awareness of the meaning of the malformation is acquired in stages [12,13]. When the diagnosis occurs at birth, the awareness is immediate because the defect is visible, but this does not mean that acceptation is immediate [14,15]. From the outset, it is important to help parents "invest in" this child who is "different" from the child they expected and hoped for; they should be helped, too, to become care auxiliaries for their infants, by way of attentive listening and information from the teams supporting them [11].

## Methods/Design

### Main hypothesis

The main hypothesis of this research is that the longer the time lapse before the first surgical intervention, the more likely are parental perceptions and feelings to upset the parent–child relationship and affect the harmonious development of the child.

### Secondary hypothesis

There are also two secondary hypotheses:

1) that the parents for whom it has been possible to give a prenatal diagnosis are better prepared to accept the waiting time

2) that with time, the negative feelings of parents in the later surgery group (3 to 6 months after birth) tend to decrease and to come into line with those of parents whose children have had an early intervention, and also that the child's distress tends to decrease.

### Main evaluation criteria

The main criteria used in this research to assess the psychological state of the infants and parental stress, as well as any possible parental psychopathology, are provided by the following measures:

1) for the children, the Alarme Détresse BéBé (ADBB) scale which measures relational withdrawal in infants. The measure is applied by a specially trained mental health professional [16]

2) the Parenting Stress Index (PSI) which enables screening for parental attitudes that could be risk factors for the development of emotional and developmental disturbances in a young child [17].

### Secondary evaluation criteria

The secondary evaluation criteria are provided by the other questionnaires selected for this research, which are:

1) the Indice de Détresse Psychologique - Enquête Santé Québec health survey (IDPESQ) [18] and the Edinburgh Post-partum Depression Scale (EPDS) [19,20] which measure the psychological state of each parent

2) the Impact On Family Scale (IOFS) [21] which assesses the family, social and financial impact of the malformation

3) Spanier's Dyadic Adjustment scale in the short version, which explores the parental couple [22,23]

4) specifically developed questionnaires collecting information on the perception of the malformation and the care team, and on the parents' present relationship with their child. These questionnaires were designed to adapt to the moment of diagnosis (antenatal or at birth).

### Experimental chronology

According to the type of CLP and the healthcare facility involved, the time lapse between diagnosis and the first surgery varies from birth to six months. Two evaluation times are planned:

· T0, when the infant is 4 months

· T1, when the child is 12 months, i.e. 6 months or more after the first surgical intervention

The choice of positioning T0 at 4 months is based on the following arguments:

· Because of the variability of the protocols used by the surgical teams in each of the centres, it is not possible to define T0 and T1 that correspond to a specific examination for all the centres;

· At this age, the children for whom the surgery occurs early have already had their operation. They can therefore be compared to children whose operation is to occur subsequently (up to 6 months), the two groups comprising roughly equivalent numbers. In a more detailed analysis, it will be possible to compare the children receiving surgery at birth with those receiving surgery at three months or more.

The choice of positioning T1 at 12 months is justified by the following argument:

· When the child is one year old, this is sufficiently distant from both birth and the first surgical intervention (by at least six months) to enable a repeat of examinations and questionnaires used in the first stage of the study. This will enable comparison of psychological perceptions of the parents and the relational development of the child between T0 and T1, as well as exploration of the question of the timing of surgery.

### Inclusion criteria

Only children with CLP are included, either isolated or familial, syndromic or nonsyndromic. Parents are included following informed consent for themselves and their child.

Two subgroups of children are also formed with respect to the time of diagnosis: 

Sub-group 1: parents having received an antenatal diagnosis

Sub-group 2: parents having discovered the diagnosis at childbirth.

For both subgroups it is in the course of the post-natal consultation that the surgical team approaches parents to enter the study, after providing all required information, and without interfering in any way with the treatment protocol.

### Non-inclusion criteria

Non-inclusion criteria are:

children with CP alone

children born before 36 weeks amenorrhoea

children whose birth weight was under 2.5 kg

children placed in foster homes

parents under legal guardianship

parents insufficiently conversant with French and/or illiterate

### Criteria for removal from the study

refusal to participate by one of the child's parents in the course of the study follow- up

the occurrence of a complication in the course of treatment and/or a serious illness requiring major specific treatment

an unexpected complication in connection with the surgical intervention

serious illness or death of one of the parents

the parents moving house outside the regions involved in the research

### Recruiting centres

Four regional centres in France are taking part in this research:

· The Strasbourg CHU Competence Centre, Pr. C. Bruant-Rodier and Dr. I. Kaufmann

· The *Centre Référent des Malformations Crânio-maxillo-faciales rares*, Lille CHU (Reference Centre), Pr. Pellerin

· The *Centre Référent des malformations rares de la face et de la cavité buccale*, APHP Paris (Reference Centre) Pr. Vazquez

· The Nancy CHU Competence Centre, Pr. Simon

These facilities were selected according to their varying lengths of waiting period between birth and the first surgical intervention (Lille: "early intervention", immediately after birth; Strasbourg: "early intervention deferred 3 months"; Hôpital Armand Trousseau (Paris) and Nancy: "Later intervention, towards 6 months". The facilities in Paris and Lille are the only Reference Centres in France, and they are distinct in particular on account of the timing of the first surgical intervention.. Their teams have international renown in the field. By way of the inclusion of a psychologist on the staff of both centres, they have long been organised to take account of the psychological impact of these malformations on parents and children. The choice of the centres in Nancy and Strasbourg enables the inclusion of two Competence Centres. These facilities have a similar organisation with a smaller staff, and do not have a psychologist permanently available.

### Data collection

The documents collected directly in the observation file for each child concern the following:

· Tests and examinations performed at T0 and T1

· Brief analysis of the individual situation of the persons present performed by the psychologist present at times T0 and T1

· Information sheet drawn up by the surgeon giving the precise type of CLP, the time when diagnosis occurred, family history, the pregnancy, care provision to the child, and also the surgeon's impression of the relationship established with the parents

· Any undesirable event

## Statistics

### Description of the statistical methods to be used

The ADBB score measured at 12 months is used as the main criterion to compare children receiving early intervention with those receiveing a later intervention. Likewise, for the parents, the PSI scores are compared between the early and later intervention groups.

A descriptive analysis of the variables will be performed. The mean, median, standard deviation and range will be provided for continuous variables, as well as proportions for categorical variables, along with 95% confidence intervals.

Scores for infant withdrawal and parental stress (ADBB and PSI in particular) will be analysed in relation to the time-lapse (two groups) before surgery (Wilcoxon-Mann- Whitney non-parametric test).

The correlations between ADBB scores and the different measures among parents (in particular PSI scores) will be studied using the Spearman correlation coefficient.

A multivariate analysis (multiple regression after transformation, where needed, of the dependent variable) will be performed to take into account the variables affecting the scores.

For all the analyses, an adjustment will be performed to take into account the type of cleft (labial cleft alone, or labial cleft with cleft palate) and whether it is unilateral or bilateral.

The analyses will be performed with SAS statistical software, release 9.2., SAS Institute Inc., Cary, NC, USA).

### Level of statistical significance

The significance level is set at 5% for all analyses. Differences corresponding to a p- value under 5% will be considered statistically significant. All tests will be two-sided.

### Dealing with missing data

Particular care will be taken to ensure that the questionnaires are satisfactorily completed, in particular where a score is to be calculated. In case of missing values, the already validated rules for replacement of missing data determined for each scale or questionnaire will be used. If necessary, for the variables considered important for the detailed analyses, multiple imputation procedures will be used.

### Number of subjects to include and justification

The number of subjects to include is estimated on the basis of a main comparison of two groups, an early intervention group and a later intervention group. For the ADBB score, the validation studies showed a standard deviation of 3.78.

Considering that a difference of 2 units in this score is clinically significant, in a two-sided test, with a type 1 error of 5%, the number of subjects required to show a difference of this magnitude with a power of 90% is 75 in each group, 150 in all. For the PSI (Parenting Stress Index), the reference studies estimated a standard deviation of 41.9 with a mean of 229. With 150 subjects, in a two-sided test, it will be possible to evidence a difference between the two groups of 10% (half a standard deviation) with a power of 90%.

### Approval

This study was approved by the Comité de Protection des Personnes Est IV of the Strasbourg teaching hospital on 18/11/2009. This approval is valid for all four of the study sites in France. The protocol conforms to the Helsinki Declaration and Good Clinical Practice guidelines of the International Conference on Harmonization. This trial is registred at ClinicalTrials.gov, Identifier: NCT00993993. This study was supported by the French Ministry of Health and it was subsequently funded accordingly.

## Discussion

### Main objectives

The aims of this research are:

1) to evaluate any affective withdrawal of infants with CLP in relation to the mental state of the parents and the waiting time before the first surgical repair intervention

2) to improve knowledge concerning the psychological effects on parents of the malformation itself, of the type of therapeutic provision in terms of time lapse between diagnosis and first surgery, of relationships with the members of the healthcare team, and of the family and social environment. In an ethical perspective, data on the time-lapse to instatement of treatment in the different protocols and the effects in each case are recorded and analysed and will serve to improve the quality of information provided during the waiting period, and the quality of the support given to the parents and the child by the healthcare team prior to the first surgical intervention. This will enable new protocols to be developed so as to minimise the psychological impact on parents as far as possible, and improve treatment of these children in the long term.

### Secondary objectives

The protocole design took account of the recommendations set out by the workshop of January 2006, entitled "Prioritizing a Research Agenda for Orofacial Clefts" [8] conducted by The National Center on Birth Defects and Developmental Disabilities and the Center for Disease Control and Prevention. The aims of the meeting were to review existing research on orofacial clefts, and to identify gaps in knowledge that need additional public health research. Of the 18 priorities defined in order of importance by this commission comprising 45 experts, those relating to the present project are 5 in number:

**Priority n°1**: Characterisation of phenotypes so as to define aetiologically homogenous categories of CLP A classification established by way of agreement among surgeons of the two tertiary referral centres is used for each of the medical files entered into the database [24,25]. The grouping of the different forms of CLP into more homogenous categories improves not only the chances of identifying risk factors, but also the prognosis for each form.

**Priority n°2**: Early screening for retarded cognitive development among children with CLP, and determination of the instruments liable to detect it. There is a need to evaluate the timing of surgery so as to ascertain whether early intervention would optimise the child's development. Beyond the immediate research objectives, and in the longer term, the formation of a cohort of patients with CLP should enable follow-up of the outcome of these young patients and their parents, in particular in periods generally considered sensitive, such as entering infant or primary school, or reaching adolescence.

**Priority n°3**: Improvement of the quality-of-life of children with CLP and their families. According to the commission, it is essential to get to know the factors that can influence quality of life, among which: support from a pluri-disciplinary team, mental balance, type and timing of surgical acts, compliance with treatment protocols, and the experience of individuals involved in the care provision.The validation of international instruments specific to children with CLP could simplify and improve the assessment of quality of life among these children. A distinction needs to be made between the perceptions of this quality of life as seen and reported by parents, and the quality of life of the children themselves, or again as perceived by the healthcare team, so as to obtain a clearer picture of the situation. This study, for the first time in this particular area, implements a specific measure assessing the parent–child relationship and integrating the state of relational withdrawal of the infant (the ADBB scale). Indeed, in the literature the parent–child relationship has always been studied by questioning the parents. All the authors who have broached this subject recognise the difficulties and the limits of these instruments which can only give one aspect of reality. The ADBB scale (developed by Antoine Guedeney, co-investigator in the present project) focuses on the child. The scale has been used in numerous international studies, but never among children with CLP [16].The PSI questionnaire has already been used for other studies on parents of children with CLP [5,26,27]. The results obtained will enable us to make comparisons with those derived fromother studies. For the present study, the IOFS (Impact On Family Scale) has been translated and validated in French. It enables the evaluation of parental quality of life, and comparison of results with those obtained by Krammer in 2007 whose study concerned families of children aged between 6 and 24 months born with orofacial clefts [28].

**Priority n°4**: The effects of the moment at which the diagnosis is received - before or after birth.Few studies have explored this theme. Better understanding of the factors liable to influence the perception of the diagnosis is required (parental stress, family support, the importance of the choice of neonatal care procedures). It is likewise important to know the manner in which parents were informed, and whether or not psychological back-up was offered. One of the secondary hypotheses of the present study is that the parents for whom a prenatal diagnosis was possible are better prepared to tolerate the waiting period until surgery.

**Priority n°5**: Care and treatment protocols for CLP.It is important to analyse the care strategies implemented for children with CLP, and also the chronology and frequencies so as to gain a better understanding of possible long-term consequences. The medical files stored in each of the participating centres give an account of the history and the chronology of the medical acts performed. Thus the protocols can be compared, in particular between the two Reference Centres in France (Lille and Paris) and the two Competence Centres (Nancy and Strasbourg).

Finally, and above all, if the results of the study point to the need for psychological support for parents of children with CLP, regardless of the timing of surgery, a list of correspondents could be made available by a reference psychiatrist belonging to the healthcare team (a recommendation of the Cleft Palate Cranio-facial Association quoted by Collet and Spetz in 2007) [29].

### Current state of the study

The study started in March 2010 and should be completed in the course of the second semester 2012. More than one hundred families of the 150 required have been included, among whom 18 have already been assessed at T0 and T1. The preliminary results cannot for the moment be reliably interpreted. However, concerning inclusions, it can be noted that certain parents refused to participate in this study. These refusals are mainly attributable to firstly the distance between home and the healthcare centre or the evaluation premises, and secondly to the repeated visits required for the care of the child, and also certainly to the reluctance of parents to confide their feelings and difficulties since the discovery of the malformation. As the study cannot intrude on the private lives of these families, it is impossible to press for agreement, especially in cases where the refusal is from one parent only. As for the evaluations, as mentioned earlier, in the literature the parent–child relationship is always studied by interviewing the parents. The choice and the relevance of the questionnaires used can be questioned. Indeed, self-administered measures are subject to caution, as parental responses can lack objectivity. The contribution of the ADBB scale for children in this age group in this instance enables the study of the parent- child relationship in a symmetrical manner, and also more objectively. The behaviour of an infant and any signs of withdrawal are unlikely to be dissimulated, which may not be the case with the parents.

## Competing interests

The authors declare that they have no competing interest.

## Funding

This work is supported by the French Ministry of Health N° IDRCB : 2009-A00640-57.

## Authors’ contributions

The project design and the different stages involved were established upstream in collaboration with the teams in the Competence and Reference Centres so as to take account of the specific features of each. BG, MPV, CBR, ADG and AG participated in the conception and design of the study and its final approval, and in the drafting and revision of the manuscript. PP, VS, CD, IK and ES participated in designing the study. MV participated in designing the biostatistical methods of the study, and in drafting and revising the manuscript. All authors have read and approved the final manuscript.

## Authors’ information

PP is the coordinator in the *Centre de Référence des Malformations Crânio-Maxillo- Faciales Rares*, and MPV is the coordinator in the *Centre de Référence des Malformations Rares de la Face et de la Cavité Buccale*. These two centres are the only Reference Centres in France for CLP. CBR and ES are respectively the coordinators of the *Centre de Compétence de la région Alsace* and the *Centre de Compétence de la région Lorraine*.

## Pre-publication history

The pre-publication history for this paper can be accessed here:

http://www.biomedcentral.com/1471-2431/12/65/prepub
